# TNF-α induces matrix metalloproteinase-9-dependent soluble intercellular adhesion molecule-1 release via TRAF2-mediated MAPKs and NF-κB activation in osteoblast-like MC3T3-E1 cells

**DOI:** 10.1186/1423-0127-21-12

**Published:** 2014-02-05

**Authors:** Chia-Lan Tsai, Wei-Chung Chen, Hsi-Lung Hsieh, Pei-Ling Chi, Li-Der Hsiao, Chuen-Mao Yang

**Affiliations:** 1 Department of Physiology and Pharmacology and Health Ageing Research Center, College of Medicine, Chang Gung University, Tao-Yuan, Taiwan; 2 Department of Nursing, Division of Basic Medical Sciences, Chang Gung University of Science and Technology, Tao-Yuan, Taiwan; 3 Department of Physiology and Pharmacology, Chang Gung University, 259 Wen-Hwa 1st Road, Kwei-San, Tao-Yuan, Taiwan

**Keywords:** TNF-α, MMP-9, Soluble ICAM-1, MAPKs, Osteoblast-like MC3T3-E1 cells

## Abstract

**Background:**

Matrix metalloproteinase-9 (MMP-9) has been shown to be induced by cytokines including TNF-α and may contribute to bone inflammatory diseases. However, the mechanisms underlying MMP-9 expression induced by TNF-α in MC3T3-E1 cells remain unclear.

**Results:**

We applied gelatin zymography, Western blot, RT-PCR, real-time PCR, selective pharmacological inhibitors of transcription (actinomycin D, Act.D), translation (cycloheximide, CHI), c-Src (PP1), MEK1/2 (U0126), p38 MAPK (SB202190), JNK1/2 (SP600125), and NF-κB (Bay11-7082), respective siRNAs transfection, promoter assay, immunofluorescence staining, and ELISA to investigate the MMP-9 expression and soluble ICAM-1 (sICAM-1) release induced by TNF-α in MC3T3-E1 cells. Here we demonstrated that TNF-α-induced MMP-9 expression was attenuated by Act.D, CHI, PP1, U0126, SB202190, SP600125, and Bay11-7082, and by the transfection with siRNAs for ERK2, p38 MAPK, and JNK2. TNF-α-stimulated TNFR1, TRAF2, and c-Src complex formation was revealed by immunoprecipitation and Western blot. Furthermore, TNF-α-stimulated NF-κB phosphorylation and translocation were blocked by Bay11-7082, but not by PP1, U0126, SB202190, or SP600125. TNF-α time-dependently induced MMP-9 promoter activity which was also inhibited by PP1, U0126, SB202190, SP600125, or Bay11-7082. Up-regulation of MMP-9 was associated with the release of sICAM-1 into the cultured medium, which was attenuated by the pretreatment with MMP-2/9i, an MMP-9 inhibitor.

**Conclusions:**

In this study, we demonstrated that TNF-α up-regulates MMP-9 expression via c-Src, MAPKs, and NF-κB pathways. In addition, TNF-α-induced MMP-9 expression may contribute to the production of sICAM-1 by MC3T3-E1 cells. The interplay between MMP-9 expression and sICAM-1 release may exert an important role in the regulation of bone inflammatory diseases.

## Background

Matrix metalloproteinases (MMPs) are a family of extracellular matrix (ECM)-degrading enzymes and induced by different stimuli including growth factors, cytokines, and tumor promoters [1]. MMPs play important roles in inflammation, tissue remodeling, angiogenesis, wound healing, and tumor invasion [2]. Furthermore, MMPs can also cleave other proteinases, latent growth factors, cell surface receptors and cell-cell adhesion molecules [3]. The important roles of MMPs have been demonstrated in bone using various approaches for ossification, remodeling, and destruction. Several literatures demonstrate that MMP-2, MMP-9, MMP-13, and MMP-14 expressed in the skeleton appear to function in ossification and remodeling [4,5]. Furthermore, MMP-2 and MMP-9 can degrade a variety of collagens including basement membrane (type IV collagen), denatured fibrillar type I collagen (gelatin), and type V collagen in osteoarticular diseases [6]. Moreover, the MMP-9 expression is highly inducible and implicated in inflammatory processes. The MMP-9 expression level has been shown to be increased in synovial effusions of rheumatoid arthritis (RA) and inflammatory arthritis (IA) samples [7]. In addition, co-culture of osteoarthritis (OA) subchondral bone osteoblasts with normal articular cartilage chondrocytes resulted in significantly increased the expression of MMP-2 and MMP-9 [8]. These studies have indicated that the expression of MMP-9 may be up-regulated during bone inflammation.

Several proinflammatory mediators, including tumor necrosis factor-α (TNF-α) have been reported to be associated with many bone functions such as resorption and inflammation. The expression of MMPs has been shown to be regulated by several extracellular stimuli such as TNF-α and IL-1β in various cell types [9,10]. Numerous studies have reported that TNF-α-induced the MMP-9 up-regulation is involved in osteoclasts during differentiation and bone destruction [11,12]. Moreover, previous studies have demonstrated that TNF-α induces the MMP-9 expression in osteoblasts and bone marrow-derived osteoprogenitor cells [13,14]. TNF-α is also elevated in the bone inflammatory patients and may exert as a major mediator in bone inflammatory diseases [15,16]. Therefore, the expression of MMP-9 induced by TNF-α may be integrated to the signaling networks that augment bone inflammation by degradation of ECM. Moreover, the expression of MMP-9 appears to be highly regulated through mitogen-activated protein kinases (MAPKs) and NF-κB in various cell types [10,17]. Cytokines such as TNF-α are reported to activate all of MAPKs including extracellular-regulated protein kinase (ERK1/2), p38 MAPK, and c-Jun-N-terminal kinase (JNK1/2) [18,19]. In cultured human chorionic trophoblast cells, TNF-α stimulates the MMP-9 secretion through the TNFR1 signaling to the MAPK pathway [20]. However, the mechanisms underlying TNF-α-stimulated MAPK activation associated with the MMP-9 gene expression in osteoblasts remain unclear. Therefore, it is needed to determine whether activation of these MAPK pathways by TNF-α is linked to the MMP-9 expression in osteoblasts. In addition, it is of interest that many of the genes regulated by these MAPK pathways are dependent on NF-κB for transcription [21] and leading to the MMP-9 gene expression at the transcriptional level [17]. In human vascular smooth muscle cells, the transcription factors NF-κB and AP-1 involved in the p42/p44 MAPK-mediated MMP-9 expression in response to TNF-α have been investigated [22]. However, the intracellular signaling mechanisms underlying the MMP-9 expression induced by TNF-α in osteoblast-like MC3T3-E1 cells are not completely characterized.

The adhesion molecule intercellular adhesion molecule-1 (ICAM-1), in addition to its membrane-associated form (mICAM-1), also exists as a soluble form (sICAM-1). In the bone microenvironment, osteoblasts play a crucial role in regulating consecutive stages of bone resorption, which include osteoclast recruitment through receptor activator of NF-κB ligand (RANKL) and mICAM-1 [23]–[25]. In clinical studies, therapy with TNF-α antagonists is able to modulate RANKL in favor of bone formation in patients with RA. Moreover, ICAM-1 belongs to the immunoglobulin superfamily which mostly serves as a counter-receptor for leukocyte integrin, lymphocyte function-associated antigen (LFA-1). Kurachi et al. have demonstrated that the interaction between LFA-1 and ICAM-1 influences the development of osteoclasts [25]. sICAM-1 is capable of binding to LFA-1 molecules [26]. Therefore, the elevated levels of sICAM-1 are thought to have immunomodulatory consequences [27]. Soluble selectins and ICAM-1 modulate neutrophil-endothelial adhesion and diapedesis *in vitro*[28]. TNF-α stimulated mICAM-1 and sICAM-1 elevation in human osteoblast-like cells isolated from OA patients [29]. The levels of sICAM-1 were also found to be elevated in RA. Furthermore, the therapeutic approaches have been taken to induce anti-inflammatory effects by blocking the ICAM-1 and TNF-α-dependent pathway with respective neutralizing antibodies [30,31]. However, the effects of the TNF-α-induced MMP-9 expression on sICAM-1 production remain unknown.

In this study, the mechanisms underlying TNF-α-induced MMP-9 expression and the effects of increased MMP-9 on MC3T3-E1 cells were investigated. We found that the activation of three MAPKs (*i.e.* ERK1/2, p38 MAPK, and JNK1/2) and NF-κB is essential for the induction of the MMP-9 gene expression in these cells. Moreover, the induction and activation of MMP-9 are important for sICAM-1 release from MC3T3-E1 cells. These results provide new insights into the mechanisms of TNF-α action that the c-Src-dependent MAPKs and IKK/NF-κB may be associated with the MMP-9 up-regulation and the sICAM-1 release from osteoblasts-like MC3T3-E1 cells.

## Methods

### Materials

Minimal essential medium-alpha (α-MEM), fetal bovine serum (FBS), and TRIzol were purchased from Invitrogen (Carlsbad, CA). Hybond C membrane and ECL Western blotting detection system were from Amersham Biosciences (Buckinghamshire, UK). Recombinant human TNF-α and the anti-TNFR1 neutralizing antibody were from R&D System (Minneapolis, MN). Luciferase assay kit was from Promega (Madison, WI). Metafectene transfection reagent was from Biontex Lab (GmbH, Planegg/Martinsried, Germany). SMARTpool RNA duplexes corresponding to Src, TRAF2, ERK2, p38 MAPK, JNK2, and scrambled #2 siRNA were from Dharmacon Research Inc (Lafayette, CO). Anti-phospho-IKKα/β, anti-phospho-NF-κB p65 (Ser^536^), anti-phospho-c-Src, anti-phospho-ERK1/2, anti-phospho-p38 MAPK, anti-phospho-JNK1/2, and anti-phospho-IκB-α antibodies were from Cell Signaling (Danver, MA). anti-NF-κB (p65), anti-lamin A, anti-TRAF2, anti-TNFR1, anti-c-Src, anti-ERK2, anti-p38, anti-JNK2, anti-IκB-α, and anti-sICAM-1 antibodies were from Santa Cruz (Santa Cruz, CA). The anti-GAPDH antibody was from Biogenesis (Boumemouth, UK). Actinomycin D (Act.D), cycloheximide (CHI), PP1, U0126, SB202190, SP600125, GM6001, MMP2/9 inhibitor, and Bay11-7082 were from Biomol (Plymouth Meeting, PA). Enzymes and other chemicals were from Sigma (St. Louis, MO).

### MC3T3-E1 osteoblastic cell culture

Murine osteoblastic cell line, MC3T3-E1, a homogeneous source of non-transformed cell, was a gift from Dr. Hyun-Mo Ryoo (Department of Oral Biochemistry, Kyungpook National University, Taegu, Korea) [32]. MC3T3-E1 cells were plated in α-MEM containing 10% (v/v) FBS at 37°C in a humidified 5% CO_2_ atmosphere. When the cultures reach confluence (4 days), cells were treated with 0.05% (w/v) trypsin/0.53 mM EDTA for 5 min at 37°C. The cell suspension was diluted with α-MEM containing 10% (v/v) FBS to a concentration of 2 × 10^5^ cells/ml. The cell suspension was plated onto (1 ml/well) 12-well culture plates and (10 ml/dish) 10-cm culture dishes for the measurement of protein expression and mRNA accumulation, respectively. Culture medium was changed after 24 h and then every 3 days.

### Gelatin zymography

MC3T3-E1 cells were plated onto 12-well culture plates and made quiescent at confluence by incubation in serum-free α-MEM for 24 h. Growth-arrested cells were incubated with TNF-α at 37°C for the indicated time intervals. When inhibitors were used, they were added 1 h prior to the application of TNF-α. The culture medium was collected and centrifuged at 14000 rpm for 5 min at 4°C to remove cells and debris, then each sample was mixed with equal amounts of (×2) non-reducible sample buffer and electrophoresed on 10% SDS-PAGE containing 1 mg/ml gelatin as the protease substrate, as previously described [33].

### Preparation of cell extracts and Western blot analysis

Growth-arrested MC3T3-E1 cells were incubated with TNF-α at 37°C for the indicated time intervals. The cells were washed, scraped, collected, lysed with ice-cold lysis buffer, and centrifuged at 45,000 *g* at 4°C for 1 h to yield the whole cell extract [34]. Samples were denatured, subjected to SDS-PAGE using a 10% running gel, and transferred to nitrocellulose membrane. Membranes were incubated overnight at 4°C with the anti-TRAF2, anti-TNFR1, anti-c-Src, anti-ERK2, anti-p38, anti-JNK2, anti-phospho-c-Src, anti-phospho-ERK1/2, anti-phospho-p38 MAPK, anti-phospho-JNK1/2, anti-phospho-c-Jun, anti-phospho-IKKα/β, anti-phospho-NF-κB (p65), anti-NF-κB (p65), anti-lamin A, anti-sICAM-1 or anti-GAPDH antibody used at a dilution of 1:2,000 in 5% (w/v) BSA in TTBS [(50 mM Tris–HCl, 150 mM NaCl, 0.05% (w/v) Tween 20, pH 7.4)]. Membranes were incubated with a 1:1500 dilution of anti-rabbit or anti-mouse horseradish peroxidase antibody for 1 h. The immunoreactive bands were detected by ECL reagents.

### Total RNA extraction, RT-PCR and real-time PCR analysis

Total RNA was isolated from MC3T3-E1 cells treated with TNF-α for the indicated time intervals with TRIzol according to the protocol of the manufacturer. RNA concentration was spectrophotometrically determined at 260 nm. First-strand cDNA synthesis was performed with 2 μg of total RNA using random hexamers as primers in a final volume of 20 μl (5 μg/μl random hexamers, 1 mM dNTPs, 2 units/μl RNasin, and 10 units/μl Moloney murine leukemia virus reverse transcriptase). The reaction was carried out at 37°C for 60 min. cDNAs encoding β-actin and MMP-9 were amplified from 3 to 5 μl of the cDNA reaction mixture using specific gene primers. Oligonucleotide primers for β-actin and MMP-9 were follow as: β-actin (514 bp): 5′-GAACCCTAAGGCCAACCGTG-3′ (sense), 5′-TGGCATAGAGGTCTTTACGG-3′ (antisense); MMP-9 (458 bp): 5′-TGTTCAGCAAGGGGCGTGTC-3′ (sense) 5′-AAACAGTCCAACAAGAAAGG-3′ (anti-sense). The amplification was performed in 35 cycles at 55°C, 1 min; 72°C, 1 min; 94°C, 1 min. After the last cycle, all samples were incubated for an additional 5 min at 72°C. The expression of β-actin was used as an internal control for the assay of a constitutively expressed gene.

Real-time PCR was performed with the TaqMan gene expression assay system, using primers and probe mixes for MMP-9 and endogenous GAPDH control genes. PCRs were performed using a 7500 Real-Time PCR System (Applied Biosystems, Foster City, CA). The primers were: 5′-(TGATGCCATTGCTGATATCCA)-3′ (sense), 5′-(CGGATCCTCAAAGGCTGAGT)-3′ (anti-sense) for MMP-9; 5′-(AACTTTGGCATCGTGGAAGG)-3′ (sense), 5′-(GTGGATGCAGGGATGATGTTC)-3′ (anti-sense) for GAPDH. Relative gene expression was determined by the ΔΔCt method, where Ct meant threshold cycle. All experiments were performed in triplicate (n = 3).

### Co-immunoprecipitation assay

Cell lysates containing 1 mg of protein were incubated with 2 μg of anti-TNFR1 antibody at 4°C for 24 h, and then 10 μl of 50% protein A-agarose beads was added and mixed at 4°C for 24 h. The immunoprecipitates were collected and washed thrice with a lysis buffer without Triton X-100. 5X Laemmli buffer was added and subjected to electrophoresis on 12% SDS-PAGE, and then blotted using the anti-TRAF2, anti-c-Src or anti-TNFR1 antibody.

### Promoter activity assay

A 710-bp (−720 to −11) segment from the 5′-promoter region of the MMP-9 gene was cloned as described [22]. Briefly, the 710-bp segment at the 5′-flanking region of the human MMP-9 gene was amplified by PCR using specific primers from the human MMP-9 gene (accession no. D10051): 5′-ACATTTGCCCGAGCTCCTGAAG-3′ (forward/SacI) and 5′-AGGGGCTGCCAGAAGCTTATGGT-3′ (reverse/HindIII). The pGL3-Basic vector, containing a polyadenylation signal upstream from the luciferase gene, was used to construct the expression vectors by subcloning PCR-amplified DNA of the MMP-9 promoter into the SacI/HindIII site of this vector. The PCR products (pGL3-WT-MMP9) were confirmed by their sizes, as determined by electrophoresis, and by DNA sequencing. Additionally, the introduction of a double-point mutation into NF-κB to generate pGL3-mt-κB-MMP9 was performed, using the following (forward) primer: mt-κB-MMP9: 5′-CTGCGGAAGACAGGCCGTTGCCCCAGTGGAATTCCC-3′ [35]. The mutants were generated using the Quick Change Site-Directed Mutagenesis Kit (Stratagene, La Jolla, CA). MMP-9-luc or κB-luc plasmid was transfected into MC3T3-E1 cells. After incubation with TNF-α (15 ng/ml), cells were collected and disrupted by sonication in a lysis buffer (25 mM Tris, pH 7.8, 2 mM EDTA, 1% Triton X-100, and 10% glycerol). After centrifugation, aliquots of the supernatants were tested for luciferase activity using the luciferase assay system. Firefly luciferase activities were standardized for β-galactosidase activity.

### Transfection with small interference RNAs

MC3T3-E1 cells were plated at 1 × 10^6^ cells/ml (1 ml/well) in 12-well culture plates for 24 h, reaching about 80% confluence. Cells were replaced with 0.4 ml of α-MEM containing 10% FBS. The DNA Metafectene reagent complex was prepared according to the instructions of the manufacturer (Biontex, Martinsried/Planegg, Germany). The amount of siRNA directed against, ERK2 (accession no. NM_138957; Dharmacon), JNK2 (accession no. NM_002752; Dharmacon), p38 (accession no. NM_139013; Dharmacon), c-Src (accession no. NM_19829; Dharmacon), TRAF2 (accession no. NM_021138; Invitrogen) or control siRNA was kept at 100 nM for each well. The DNA Metafectene complex (0.1 ml) was added to each well and then incubated at 37°C for 24 h. The cells were washed twice with PBS and maintained in α-MEM containing 1% FBS for 72 h before treatment with TNF-α for the indicated time intervals.

### NF-κB translocation

MC3T3-E1 cells were seeded in a 10-cm dish. After they reached 90% confluence, cells were starved for 24 h in serum-free α-MEM medium. After stimulation with 15 ng/ml TNF-α for various time intervals, and when inhibitors were used, they were added 1 h prior to the application of TNF-α. As previously described [34], the cells were washed once with ice-cold PBS, 200 μl of homogenization buffer A (20 mM Tris–HCl, pH 8.0, 10 mM EGTA, 2 mM EDTA, 2 mM dithiothreitol, 1 mM phenylmethylsulfonyl fluoride, 25 μg/ml aprotinin, 10 μg/ml leupeptin) was added to each dish, and the cells were scraped into a 1.5-ml Eppendorf vial. The suspension was sonicated for 10 s at the output 4 with a sonicator (Misonix Inc., Farmingdale, NY) and centrifuged at 8000 rpm at 4°C for 5 min. The pellet was collected as the nuclear fraction. The pellet was resuspended in 300 μl of homogenization buffer B (1% Triton X-100 in buffer A) and sonicated for 10 sec. The supernatant was centrifuged at 15000 rpm at 4°C for 15 min. The supernatant was collected as a cytosolic fraction and the pellet as a membrane fraction. Protein concentration was determined by using BCA reagents. Samples (30 μg protein) were denatured and subjected to SDS-PAGE using a 10% (w/v) running gel. Proteins were transferred to a nitrocellulose membrane and the membranes were successively incubated at room temperature with 1% (w/v) BSA in TTBS for 1 h. The translocation of NF-κB was identified and quantified by Western blot using the anti-phospho-IκB-α, IκB-α, and NF-κB (p65) antibodies. The immunoreactive bands were detected by ECL reagents.

### Immunofluorescent staining

MC3T3-E1 cells were plated on 6-well culture plates with coverslips. Cells were further cultured in serum-free α-MEM for 24 h and treated with 15 ng/ml TNF-α for various time intervals. When inhibitors were used, they were added 1 h prior to the application of TNF-α. After washing twice with ice-cold PBS, the cells were fixed with 4% (w/v) paraformaldehyde in PBS for 30 min, and then permeabilized with 0.3% Triton X-100 in PBS for 15 min. The staining was performed by incubating with 10% normal goat serum in PBS for 30 min followed by incubating with the primary NF-κB (p65) antibody (1:100 dilution) for 1 h in PBS with 1% BSA, washing thrice with PBS, incubating for 1 h with fluorescein isothiocyanate (FITC)-conjugated anti-rabbit antibody (1:100 dilution) in PBS with 1% BSA, washing thrice with PBS, and finally mounting with aqueous mounting medium. The cell nucleus was stained by DAPI. The images were observed under a fluorescence microscope (Zeiss, Axiovert 200 M).

### Measurement of sICAM-1 generation

sICAM-1 released into the culture media of MC3T3-E1 cells was collected and detected by using an ELISA kit according to the manufacturer’s instructions (R&D System, Minneapolis, MN).

### Statistical analysis of data

Concentration-effect curves were fitted and estimated by using the GraphPad Prism Program (GraphPad, San Diego, CA). Data were expressed as mean ± S.E.M. and analyzed by one-way ANOVA followed with Tukey’s post-hoc test. *P* < 0.05 was considered significant.

## Results

### TNF-α induces MMP-9 expression in MC3T3-E1 cells

**Figure 1:**
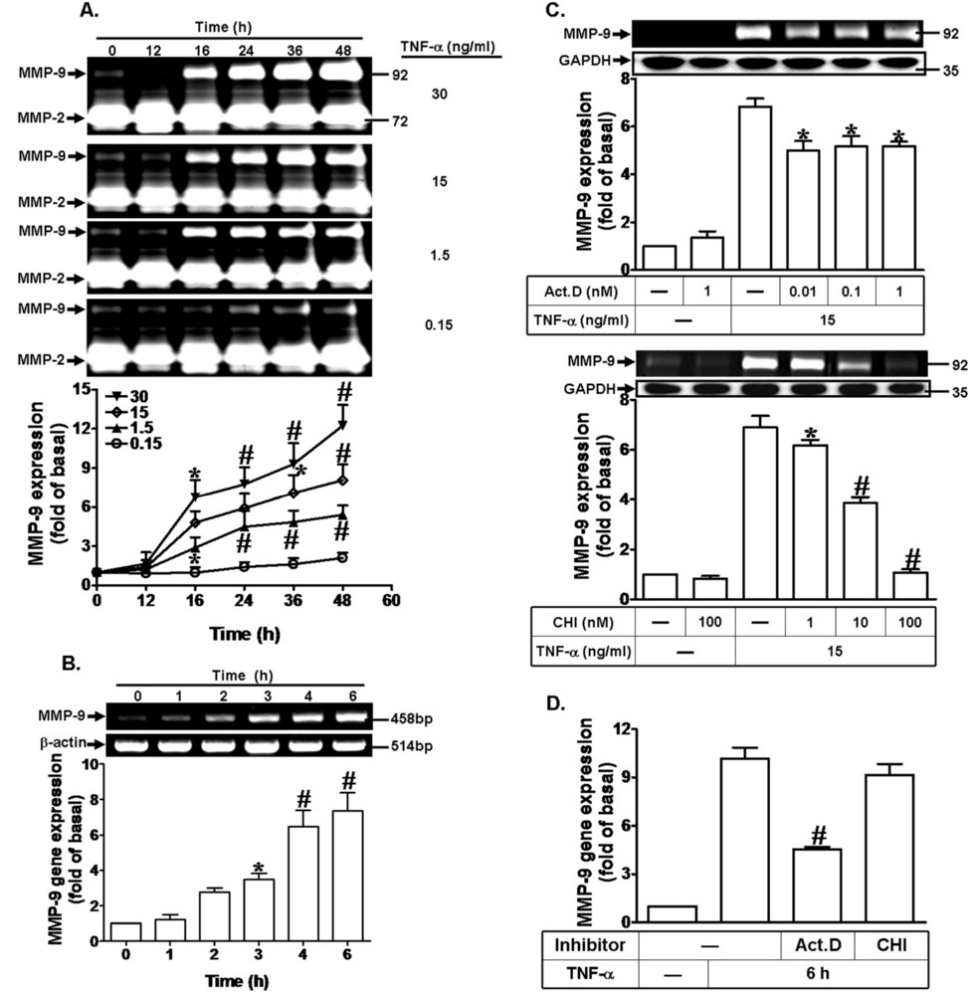
**TNF-α induces MMP-9 expression through transcription and translation levels. (A)** MC3T3-E1 cells were incubated with various concentrations of TNF-α for the indicated time intervals. The conditioned media were collected and analyzed by gelatin zymography. The proteolytic activities of MMP-9 and MMP-2 were manifested as horizontal white bands on a blue background, and the expression of MMP-2 served as an internal control. **(B)** Cells were treated with TNF-α (15 ng/ml) for various time intervals. The total RNA were collected and analyzed by RT-PCR and real-time PCR. **(C)** Growth-arrested cells were pretreated with various concentrations of either Act. D or CHI for 1 h and then incubated with TNF-α (15 ng/ml) for 24 h. The conditioned media were collected and assayed by gelatin zymography. The cell lysates were analyzed by Western blot to determine the expression of GAPDH as an internal control. **(D)** Cells were pretreated with Act.D (1 nM) or CHI (100 nM) for 1 h and then incubated with TNF-α (15 ng/ml) for 6 h. The isolated RNA samples were analyzed for the level of MMP-9 mRNA by real-time PCR. Data are expressed as mean±SEM of three independent experiments. ^*^P < 0.05, ^#^*P* <0.01, as compared to the cells treated with vehicle **(A,B)** and TNF-α alone **(C,D)**.

TNF-α has been shown to induce the expression of MMP-9 in human osteoblasts, osteoprogenitors, and mesenchymal stem cells [13,36]. To determine the effect of TNF-α on MMP-9 expression, MC3T3-E1 cells were incubated with various concentrations of TNF-α for the indicated time intervals. The conditioned media were collected to determine the MMP-9 expression activity by gelatin zymography. As shown in Figure 1A, the conditioned media from MC3T3-E1 cells displayed proteolytic activity at 110 kDa, corresponding to the pro-form of mouse MMP-9, and TNF-α induced proMMP-9 expression in a time- and concentration-dependent manner. There was a significant increase within 16 h and a maximal increase was achieved by 36–48 h during the period of observation. In contrast, TNF-α had no effect on MMP-2 expression which served as an internal control. To further examine whether the increase in MMP-9 expression induced by TNF-α results from an increase of MMP-9 mRNA expression, MC3T3-E1 cells were incubated with 15 ng/ml TNF-α for the indicated time intervals. The levels of MMP-9 mRNA were determined by RT-PCR and real-time PCR. As shown in Figure 1B, TNF-α time-dependently induced the expression of MMP-9 mRNA, a significant increase within 4 h and reached a peak by 6 h. These data suggested that TNF-α induces MMP-9 expression via increasing mRNA and protein levels in MC3T3-E1 cells. We further investigated whether TNF-α-induced MMP-9 expression is mediated through transcription and translation, a transcription inhibitor Act.D and a translation inhibitor CHI were used for these purposes. The data showed that the pretreatment with either Act.D or CHI concentration-dependently blocked TNF-α-induced MMP-9 expression determined by gelatin zymography (Figure 1C), suggesting that TNF-α-induced proMMP-9 expression occurs at both transcriptional and translational levels. Additionally, TNF-α-induced MMP-9 mRNA expression was inhibited by Act.D, but not CHI, revealed by real-time PCR (Figure 1D). These results indicated that TNF-α induces MMP-9 expression via both transcription and translation levels in MC3T3-E1 cells.

### Involvement of TNFR1-dependent pathway in TNF-α-induced MMP-9 expression

**Figure 2:**
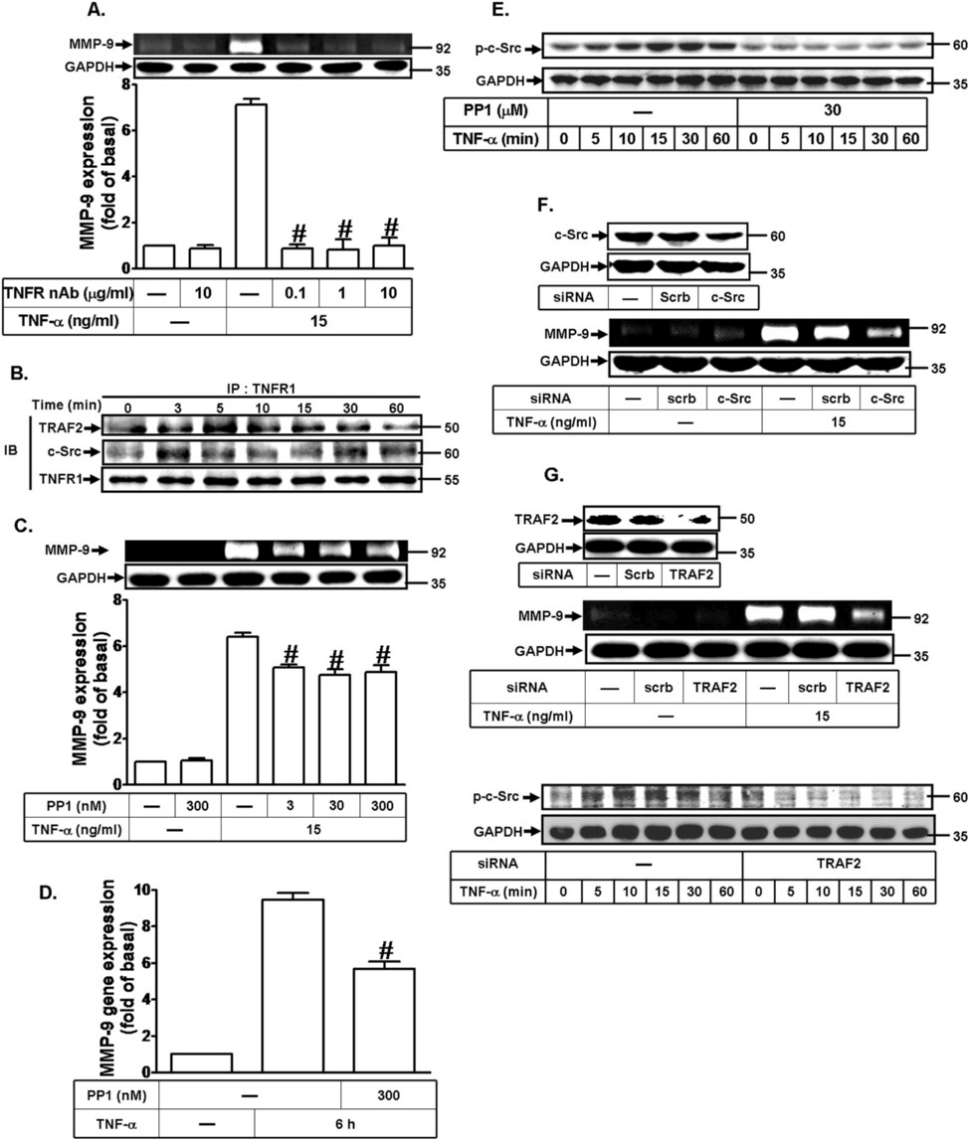
**TNF-α induces MMP-9 expression via TNFR1-dependent c-Src cascade. (A)** Cells were pretreated with various concentrations of TNF-α receptor antibody (TNFR Ab) for 1 h and then incubated with TNF-α (15 ng/ml) for 24 h. **(B)** MC3T3-E1 cells were incubated with TNF-α (15 ng/ml) for the indicated time intervals, and the protein-protein interaction was determined by immunoprecipitation (IP) and Western blot using the antibodies as indicated. **(C,D)** Cells were treated with TNF-α (15 ng/ml) for **(C)** 24 h or **(D)** 6 h in the absence or presence of PP1. **(D)** The isolated RNA samples were analyzed for the levels of MMP-9 mRNA by real-time PCR. Data are expressed as mean±SEM of three independent experiments. ^*^P < 0.05; ^#^*P* < 0.01, as compared to the cells incubated with TNF-α alone. **(E,G)** Cells were pretreated with or without PP1 (30 μM) for 1 h or transfected with TRAF2 siRNA and then stimulated with TNF-α for the indicated time intervals. Phosphorylation of c-Src was determined by Western blot using an anti-phospho-c-Src antibody. **(F,G)** Cells were transfected with c-Src siRNA or TRAF2 siRNA for 24 h and then incubated with TNF-α (15 ng/ml) for 24 h. **(A,C,F,G)** MMP-9 expression was determined as described in Figure 1. The cell lysates were determined by Western blot using an anti-c-Src, anti-TRAF2 or anti-GAPDH antibody.

TNF receptor l (TNFR1) and TNF receptor-associated factor 2 (TRAF2) generate distinct signals by TNF-α for the induction of differently biological responses in many cell types. Recent evidences suggest that MMP-9 expression was markedly suppressed in TNFR1 KO mice as compared to wild-type mice [37]. Previously, Lee et al. have demonstrated that TNF-α triggered the association between TNFR1 and TRAF2 to induce the MMP-9 expression in A549 cells [38]. In addition, TNFR1 may associate with kinases such as c-Src to engage signaling pathways, activate transcription factors, and modulate gene expression in various cell types [39]. Therefore, to investigate whether TNF-α induces MMP-9 expression via TNFR1, a neutralizing TNFR antibody was used. As shown in Figure 2A, the pretreatment with the TNFR antibody attenuated TNF-α-induced MMP-9 expression in a concentration-dependent manner. Moreover, to demonstrate whether TNFR1-relative proteins are involved in this response, the cell lysates were immunoprecipitated using an anti-TNFR1 antibody and analyzed by Western blot. As shown in Figure 2B, TNF-α stimulated association of TNFR1, TRAF2, and c-Src in a time-dependent manner. There was a significant increase of TRAF2 and c-Src within 3–5 min during the period of observation. Furthermore, the pretreatment with a c-Src inhibitor PP1 attenuated TNF-α-induced MMP-9 expression in a concentration-dependent manner (Figure 2C), confirming that TNF-α-induced MMP-9 expression is mediated through c-Src. Similarly, pretreatment with PP1 also inhibited TNF-α-induced MMP-9 mRNA expression (Figure 2D). Moreover, we investigated whether TNF-α-induced c-Src activation, c-Src phosphorylation was determined by Western blot using anti-phospho-c-Src antibody and transfection with siRNA for TRAF2. As shown in Figure 2E and G, TNF-α time-dependently stimulated c-Src phosphorylation with a significant increase within 10 min and a maximal response within 15 min. Moreover, pretreatment with PP1 (30 μM) and siRNA for TRAF2 significantly attenuated c-Src phosphorylation in response to TNF-α during the period of observation. To further confirm the role of c-Src or TRAF2 in TNF-α-induced MMP-9 expression, cells were transfected with c-Src or TRAF2 siRNA and then incubated with TNF-α for 24 h. Transfection with c-Src or TRAF2 siRNA down-regulated the total c-Src or TRAF2 protein expression and attenuated TNF-α-induced MMP-9 expression (Figure 2F and H). These results suggested that TNF-α-induced MMP-9 expression is mediated through TNFR1-dependent TRAF2 linking to c-Src cascade in MC3T3-E1 cells.

### TNF-α induces MMP-9 expression via ERK1/2 phosphorylation

**Figure 3:**
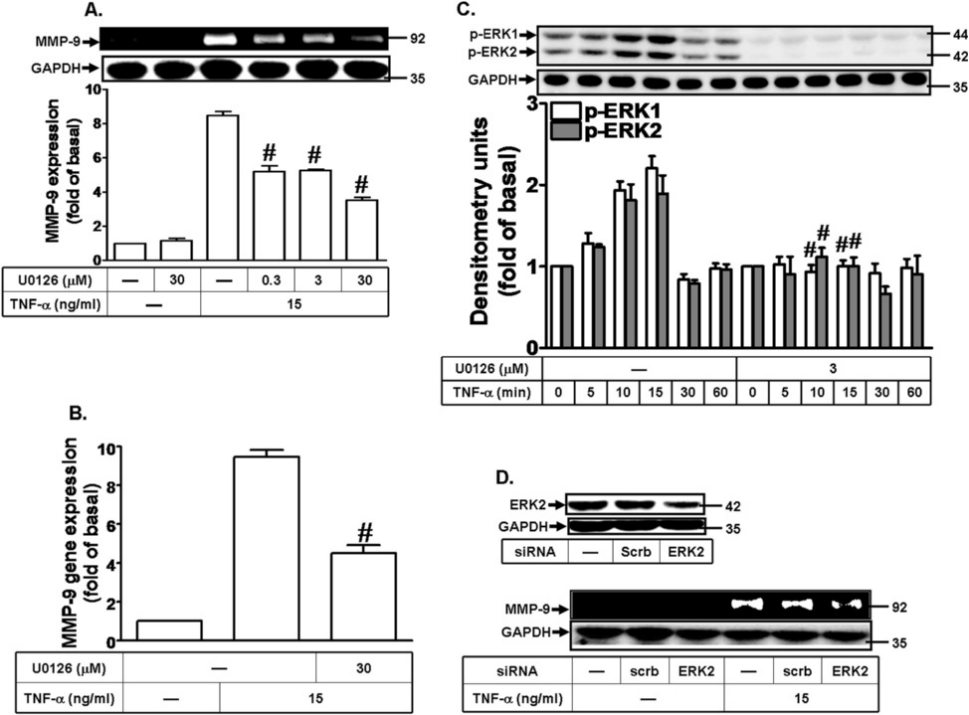
**TNF-α induces MMP-9 expression via ERK1/2 phosphorylation. (A)** Cells were pretreated with U0126 for 1 h and then incubated with TNF-α (15 ng/ml) for 24 h. **(B)** Cells were pretreated with U0126 (3 μM) for 1 h and then incubated with TNF-α (15 ng/ml) for 6 h. The isolated RNA samples were analyzed for the levels of MMP-9 mRNA by real-time PCR. **(C)** Cells were pretreated with or without U0126 (3 μM) for 1 h and then stimulated with TNF-α for the indicated time intervals. Phosphorylation of ERK1/2 was determined by Western blot using an anti-phospho-ERK1/2 antibody. Data are expressed as mean±SEM of three independent experiments. ^#^*P* < 0.01, as compare with the cells treated with TNF-α alone. **(D)** Cells were transfected with ERK2 siRNA for 24 h and then incubated with TNF-α (15 ng/ml) for 24 h. **(A,D)** MMP-9 expression was determined as described in Figure 1. The cell lysates were determined by Western blot using an anti-ERK2 or anti-GAPDH antibody.

MAPKs, including ERK1/2, p38 MAPK, and JNK1/2, can regulate expression of several genes through activation of downstream kinases or nuclear proteins. Previous study has demonstrated that TNF-α induces MMP-9 expression via p42/p44 MAPK and JNK1/2 in A549 cells [40]. Here, to determine whether ERK1/2 activation is involved in TNF-α-induced MMP-9 expression in MC3T3-E1 cells, a pharmacological inhibitor of MEK1/2 (U0126) was used. Pretreatment with U0126 attenuated TNF-α-induced MMP-9 protein expression in a concentration-dependent manner (Figure 3A) and MMP-9 mRNA expression (Figure 3B), suggesting that MEK1/2-ERK1/2 is involved in TNF-α-induced MMP-9 expression. To further determine whether phosphorylation of ERK1/2 is necessary for TNF-α-induced MMP-9 expression, activation of ERK1/2 was assayed by Western blot using an antibody specific for the phosphorylated, active forms of ERK1/2. As shown in Figure 3C, TNF-α time-dependently stimulated ERK1/2 phosphorylation with a significant increase within 10 min and a maximal response within 15 min in MC3T3-E1 cells. Pretreatment with U0126 (3 μM) significantly attenuated TNF-α-induced ERK1/2 phosphorylation during the period of observation. These results suggested a link between activation of the ERK1/2 pathway and up-regulation of MMP-9 induced by TNF-α in MC3T3-E1 cells. To further confirm the role of ERK1/2 in TNF-α-induced MMP-9 expression, cells were transfected with ERK2 siRNA and then incubated with TNF-α for 24 h. Transfection with ERK2 siRNA down-regulated the total ERK2 protein expression and attenuated TNF-α-induced MMP-9 expression (Figure 3D). These data suggested that TNF-α-induced MMP-9 expression is mediated through a MEK1/2-ERK1/2 pathway in MC3T3-E1 cells.

### TNF-α-induced MMP-9 expression via p38 MAPK phosphorylation

**Figure 4:**
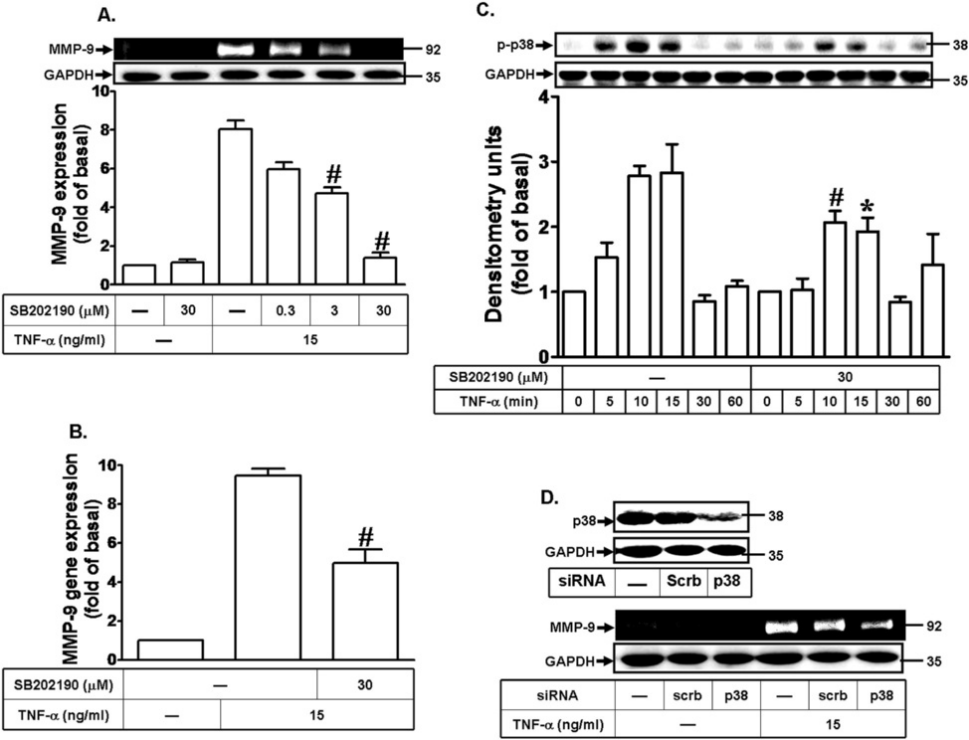
**Involvement of p38 MAPK phosphorylation in TNF-α-induced MMP-9 expression. (A)** Cells were pretreated with SB202190 for 1 h and then incubated with TNF-α (15 ng/ml) for 24 h. **(B)** Cells were pretreated with SB202190 (30 μM) for 1 h and then incubated with TNF-α (15 ng/ml) for 6 h. The isolated RNA samples were analyzed for the levels of MMP-9 mRNA by real-time PCR. Data are expressed as mean±SEM of three independent experiments. ^*^P < 0.05; ^#^*P* < 0.01, as compare to the cells treated with TNF-α alone. **(C)** Cells were pretreated with or without SB202190 (30 μM) for 1 h and then stimulated with TNF-α for the indicated time intervals. Phosphorylation of p38 MAPK was determined by Western blot using an anti-phospho-p38 MAPK antibody. **(D)** Cells were transfected with p38 siRNA for 24 h and then incubated with TNF-α (15 ng/ml) for 24 h. **(A,D)** MMP-9 expression was determined as described in Figure 1. The cell lysates were determined by Western blot using an anti-p38 MAPK or anti-GAPDH antibody.

To determine whether p38 MAPK is involved in TNF-α-induced MMP-9 expression, a p38 MAPK inhibitor (SB202190) was used. As shown in Figures 4A and B, the pretreatment with SB202190 significantly attenuated TNF-α-induced MMP-9 expression in a concentration-dependent manner and mRNA expression revealed by gelatin zymography and real-time PCR, respectively. To further determine whether TNF-α stimulates p38 MAPK activation, the phosphorylation of p38 MAPK was assayed by Western blot using an antibody specific for the phosphorylated, active form of p38 MAPK. As shown in Figure 4C, TNF-α time-dependently stimulated phosphorylation of p38 MAPK in MC3T3-E1 cells. A maximal response was obtained within 10 min and declined to the basal level within 30 min. Moreover, pretreatment with SB202190 (particularly 30 μM) attenuated TNF-α-stimulated p38 MAPK phosphorylation during the period of observation. These results suggested a link between phosphorylation of p38 MAPK and up-regulation of MMP-9 induced by TNF-α in MC3T3-E1 cells. To further ensure the involvement of p38 MAPK in TNF-α-induced MMP-9 expression, cells were transfected with p38 MAPK siRNA. The results showed that transfection with p38 MAPK siRNA down-regulated the total p38 protein expression and attenuated TNF-α-induced MMP-9 expression (Figure 4D). These data suggested that TNF-α-induced MMP-9 expression is mediated through a p38 MAPK pathway in MC3T3-E1 cells.

### TNF-α induces MMP-9 expression via JNK1/2 phosphorylation

**Figure 5:**
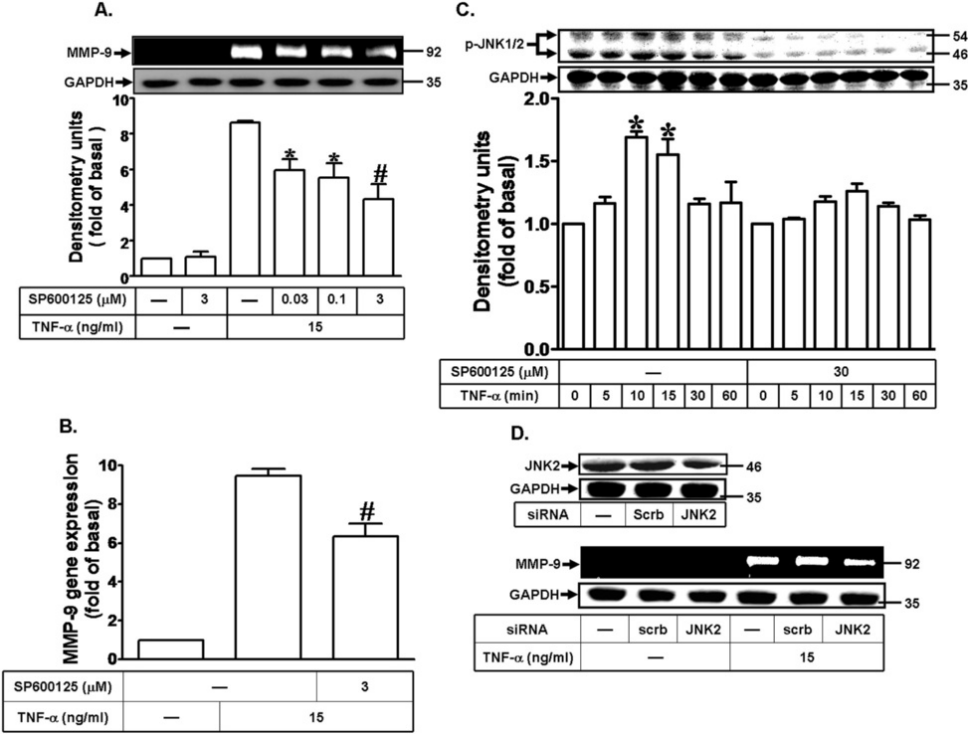
**TNF-α induces MMP-9 expression via JNK1/2 phosphorylation. (A)** Cells were pretreated with SP600125 for 1 h and then incubated with TNF-α (15 ng/ml) for 24 h. **(B)** Cells were pretreated with SP600125 (3 μM) for 1 h and then incubated with TNF-α (15 ng/ml) for 6 h. The isolated RNA samples were analyzed for the levels of MMP-9 mRNA by real-time PCR. **(C)** Cells were pretreated without or with SP600125 for 1 h and then incubated with TNF-α (15 ng/ml) for various time intervals. The cell lysates were analyzed by Western blot using an anti-phospho-JNK1/2 antibody or anti-GAPDH (as an internal control) antibody. Data are expressed as mean±SEM of three independent experiments. ^#^*P* < 0.01, as compare to the cells treated with TNF-α alone. *P < 0.05, as compare to the cells treated with TNF-α alone **(A)** or vehicle **(C). (D)** Cells were transfected with JNK2 siRNA for 24 h and then incubated with TNF-α (15 ng/ml) for 24 h. **(A,D)** MMP-9 expression was determined as described in Figure 1. The cell lysates were determined by Western blot using an anti-JNK2 or anti-GAPDH antibody.

In addition, to determine whether the activation of JNK1/2 is also involved in TNF-α-induced MMP-9 expression, a pharmacological inhibitor of JNK1/2 SP600125 was used. As shown in Figures 5A and B, the pretreatment with SP600125 attenuated TNF-α-induced MMP-9 expression in a concentration-dependent manner and mRNA expression, revealed by zymography and real-time PCR. We further investigated whether JNK1/2 phosphorylation participates in TNF-α-induced MMP-9 expression in MC3T3-E1 cells, activation of JNK1/2 was assayed by Western blotting using an antibody specific for the phosphorylated, active forms of JNK1/2. We found that TNF-α time-dependently stimulated JNK1/2 phosphorylation with a significant increase within 10 min and a maximal response within 60 min in MC3T3-E1 cells (Figure 5C), which was attenuated by the pretreatment with SP600125 during the period of observation. Moreover, we confirmd the role of JNK1/2 in TNF-α-induced MMP-9 expression, cells were transfected with a JNK2 siRNA. The data showed that transfection with JNK2 siRNA down-regulated the total JNK2 protein expression and attenuated TNF-α-induced MMP-9 expression (Figure 5D). These data suggested that TNF-α-induced MMP-9 expression is mediated through a JNK1/2 pathway in MC3T3-E1 cells.

### NF-κB is required for TNF-α-induced MMP-9 expression

**Figure 6:**
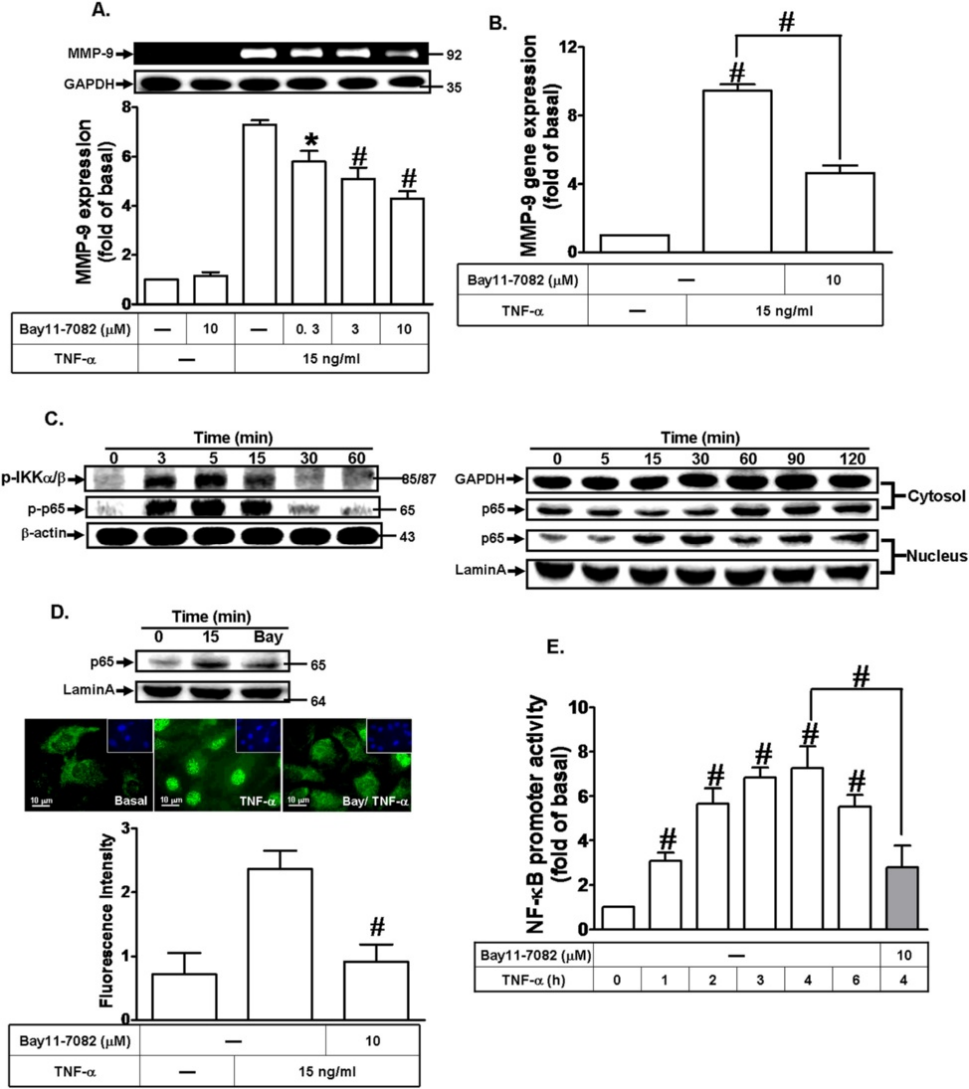
**NF-κB is required for TNF-α-induced MMP-9 expression. (A)** Cells were pretreated with Bay11-7082 for 1 h and then incubated with TNF-α (15 ng/ml) for 24 h. MMP-9 expression was determined as described in Figure 1. **(B)** Cells were pretreated with Bay11-7082 (10 μM) for 1 h and then incubated with TNF-α (15 ng/ml) for 6 h. The isolated RNA samples were analyzed for the levels of MMP-9 mRNA by real-time PCR. **(C)** Cells were incubated with TNF-α (15 ng/ml) for the indicated time intervals. The cell lysates were analyzed by Western blot using an anti-phospho-IKKα/β, anti-phospho-p65, or anti-β-actin antibody (left part). The cytosolic and nuclear fractions were analyzed by Western blot using an anti-NF-κB (p65), anti-Lamin A, and anti-GAPDH antibody (right part). **(D)** Cells were pretreated without or with Bay11-7082 (10 μM) for 1 h and then stimulated with TNF-α (15 ng/ml) for 15 min. The nuclear fraction was analyzed by Western blot using an anti-NF-κB (p65) and anti-Lamin A antibody. The translocation of p65 NF-κB was also observed by immunofluorescence staining (middle part) and the histogram of p65 translocation (lower part). **(E)** Cells were transiently transfected with NF-κB-Luc construct and then incubated with TNF-α (15 ng/ml) for the indicated time intervals in the absence or presence of Bay11-7082 (10 μM). The cell lysates were collected and determined the luciferase activity. Data are expressed as mean±SEM of three independent experiments. ^#^*P* < 0.01, as compare to the cells treated with TNF-α alone. *P < 0.05, as compare to the cells treated with TNF-α **(A,B,E)** or vehicle alone **(E)**.

Inflammatory responses following stimulation by TNF-α are highly dependent on activation of the transcription factor NF-κB. Moreover, NF-κB is one of the major mediators of the intracellular functions of TNF-α. Therefore, we investigated whether the involvement of NF-κB activation in TNF-α-induced MMP-9 expression in MC3T3-E1 cells, an NF-κB pharmacological inhibitor Bay11-7082 was used. As shown in Figure 6A and B, the pretreatment with Bay11-7082 caused an attenuation of TNF-α-induced MMP-9 protein in a concentration-dependent manner and mRNA expression, revealed by zymography and real-time PCR, respectively. We further determined whether TNF-α induces MMP-9 expression through activation of an NF-κB upstream molecule IKKα/β and p65 NF-κB phosphorylation, the phosphorylation of IKKα/β and p65 was assayed by Western blotting using an antibody specific for the phosphorylated, active forms of IKKα/β and p65, respectively. As shown in Figure 6C (left panel), TNF-α time-dependently stimulated phosphorylation of IKKα/β and p65 in MC3T3-E1 cells.

A significant response was obtained within 5–15 min and declined to the basal level within 30 min. To investigate whether TNF-α stimulates translocation of p65 NF-κB, the cytosolic and nuclear fractions were used to determine the p65 translocation by Western blotting using an anti-p65 antibody. The data showed that TNF-α time-dependently induced translocation of the p65 subunit of NF-κB into nucleus with a significant increase within 15–30 min (Figure 6C, right panel). Pretreatment with Bay11-7082 (10 μM) attenuated TNF-α-stimulated p65 NF-κB translocation revealed by Western blotting and immunofluorescence staining analyses (Figure 6D), suggesting that NF-κB is essential for TNF-α-induced MMP-9 expression in MC3T3-E1 cells. Moreover, to determine whether TNF-α stimulates NF-κB transcriptional activity, a κB-luciferase reporter construct was used. As shown in Figure 6E, TNF-α stimulated a time-dependent NF-κB transcriptional activity with a maximal response by 4 h. Pretreatment with Bay11-7082 significantly inhibited TNF-α-induced NF-κB transcriptional activity. These results demonstrated that NF-κB is required for TNF-α-induced MMP-9 expression in MC3T3-E1 cells.

### TNF-α stimulates two independent pathways: c-Src-dependent MAPKs and NF-κB-dependent cascades in MC3T3-E1 cells

**Figure 7:**
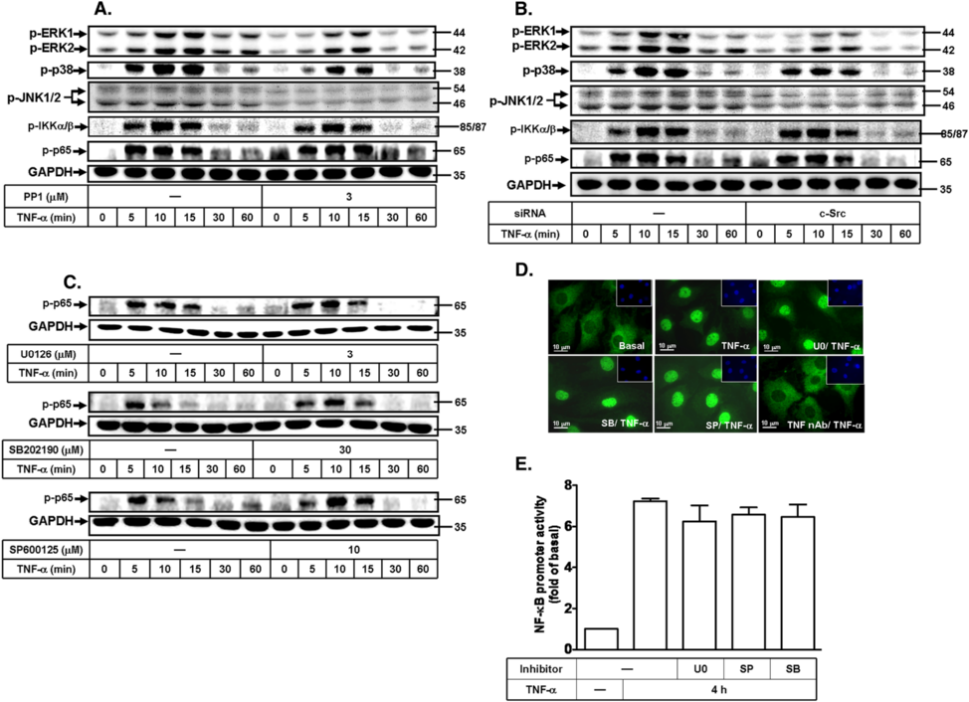
**TNF-α stimulates c-Src-dependent MAPKs and NF-κB-dependent cascades in MC3T3-E1 cells.** MC3T3-E1 cells were pretreated with 3 μM PP1 **(A)** transfected with c-Src siRNA **(B)** or pretreated with **(C)** U0126 (3 μM), SB202190 (3 μM), or SP600125 (3 μM) for 1 h, and then stimulated with TNF-α (15 ng/ml) for the indicated time intervals. The cell lysates were analyzed by Western blot using an anti-phospho-ERK1/2, anti-phospho-p38 MAPK, anti-phospho-JNK1/2, anti-phospho-IKKα/β, anti-phospho-p65, or anti-GAPDH (as a control) antibody. **(D)** Cells were pretreated with U0126 (3 μM), SB202190 (3 μM), SP600125 (3 μM), or TNF-α receptor 1 neutralized antibody (TNFR nAb) for 1 h and then stimulated with TNF-α (15 ng/ml) for 15 min. The translocation of p65 NF-κB was observed by immunofluorescence staining. **(E)** Cells were transfected with NF-κB-Luc construct, pretreated with U0126 (3 μM), SB202190 (3 μM), or SP600125 (3 μM) for 1 h, and then incubated with TNF-α (15 ng/ml) for 4 h. The cell lysates were collected and determined NF-κB-Luc activity. Similar results were obtained in three independent experiments.

According to the above data, we have demonstrated that TNF-α induced MMP-9 expression via activation of c-Src, ERK1/2, p38 MAPK, JNK1/2, and NF-κB in MC3T3-E1 cells. It would be important to determine the relationship among these molecules, including c-Src, MAPKs, and NF-κB in the responses. Cells were pretreated with the inhibitor of c-Src (PP1), MEK1/2 (U0126), p38 MAPK (SB202190), or JNK1/2 (SP600125) for 1 h and then stimulated with TNF-α for the indicated time intervals. Phosphorylation of ERK1/2, p38 MAPK, JNK1/2, IKKα/β and p65 was assayed by Western blotting. As shown in Figure 7A, TNF-α-stimulated phosphorylation of ERK1/2, p38 MAPK, and JNK1/2, but not IKKα/β and p65 was significantly attenuated by the pretreatment with PP1 during the period of observation. Moreover, PP1 has inhibitory effects on not only c-Src but also other Src family kinases. Therefore, MC3T3-E1 cells were transfected with c-Src siRNA to confirm whether MAPKs and the IKK/NF-κB pathway are inhibited by c-Src knockdown. The data were correlated with Figure 7A, TNF-α-stimulated phosphorylation of ERK1/2, p38 MAPK, and JNK1/2, but not IKKα/β and p65 was significantly attenuated by transfection with siRNA of c-Src during the period of observation (Figure 7B). The data demonstrated that TNF-α-stimulated phosphorylation of ERK1/2, p38 MAPK, and JNK1/2 is dependent on c-Src activation. TNF-α-stimulated p65 NF-κB activation was independent of c-Src. Moreover, we found that TNF-α-stimulated p65 phosphorylation and translocation was not significantly inhibited by the pretreatment with U0126, SB202190, or SP600125 determined by Western blotting during the period of observation (Figure 7C) and immunofluorescence staining of p65 NF-κB (Figure 7D). Subsequently, we also demonstrated that TNF-α-stimulated NF-κB transcriptional activity is independent of these MAPKs, revealed by NF-κB-luciferase reporter assay (Figure 7E). These data demonstrated that TNF-α induced MMP-9 expression via two independent pathways, including c-Src-dependent MAPKs and c-Src-independent IKK/NF-κB cascades in MC3T3-E1 cells.

### The NF-κB element is important for TNF-α-induced MMP-9 gene promoter activation

**Figure 8:**
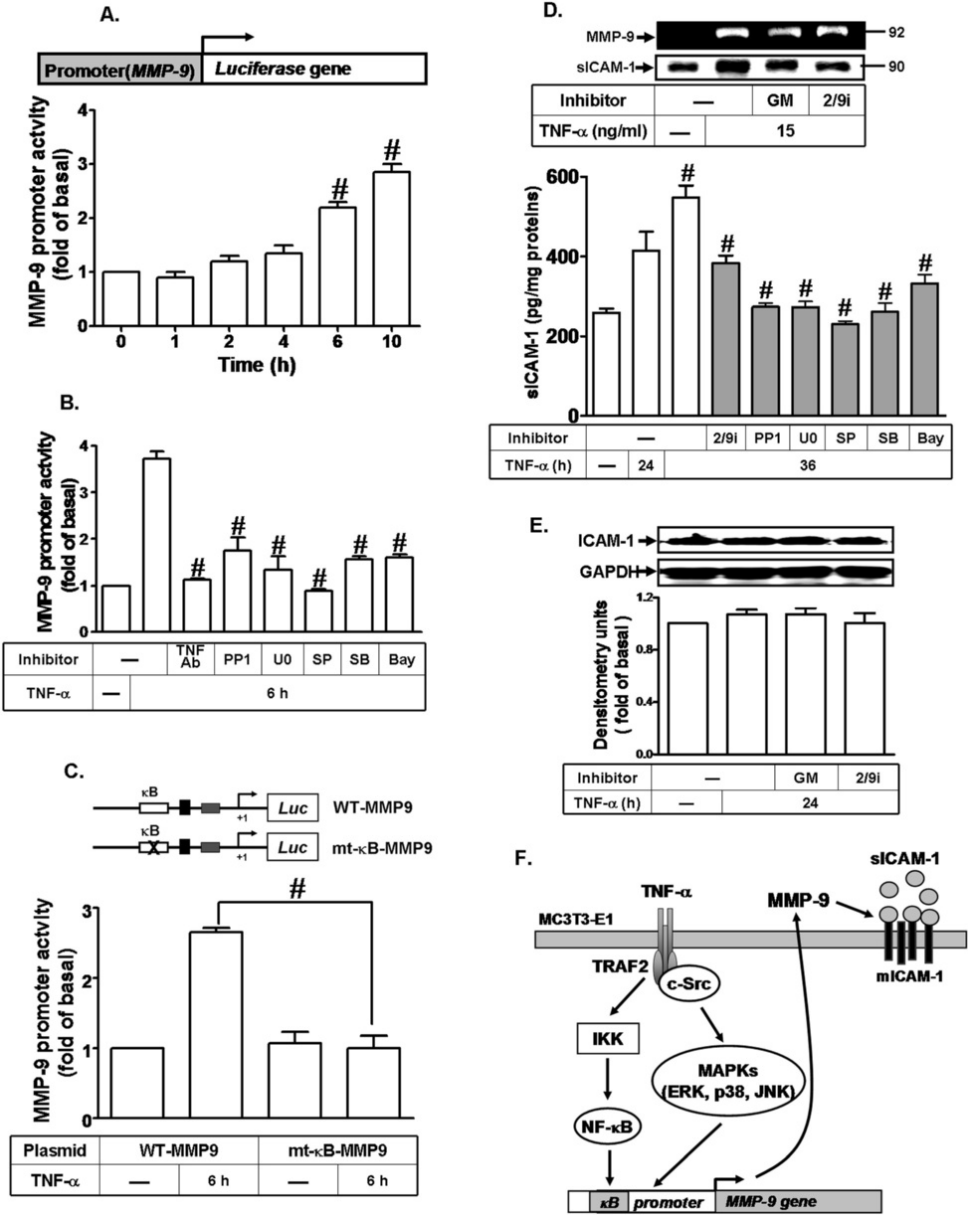
**TNF-α-induced MMP-9 expression is mediated through the NF-κB element in MMP-9 promoter leading to soluble ICAM-1 release. (A)** Cells were transiently transfected with a wild-type MMP-9 promoter-luciferase reporter construct (WT-MMP9), and then incubated with TNF-α for the indicated time intervals. **(B)** The WT-MMP9 transfected cells were pretreated with anti-TNFR1 neutralizing antibody (TNFR-Ab, 10 μg/ml), PP1 (3 μM), U0126 (3 μM), SB202190 (3 μM), SP600125 (3 μM), and Bay11-7082 (10 μM) for 1 h and then incubated with 15 ng/ml TNF-α for 6 h. **(C)** Cells were transfected with WT-MMP9 or mt-κB-MMP9 for 24 h and then incubated with TNF-α (15 ng/ml) for 6 h. The cell lysates were collected and determined the luciferase activity. **(D,E)** Cells were pretreated with GM6001 or MMP2/9i for 1 h and then incubated with TNF-α (15 ng/ml) for 24 h. **(D, upper panel)** Conditioned media were collected and analyzed by gelatin zymography to determine the MMP-9 expression. The conditioned media were analyzed by trichloroacetic acid-protein precipitation and Western blot using an anti-sICAM-1 antibody. **(E)** The cell lysates were analyzed by Western blot to determine the expression of ICAM-1. **(D, lower panel)** Cells were pretreated with MMP-2/9i (10 μM), PP1 (10 μM), U0126 (10 μM), SB202190 (10 μM), SP600125 (10 μM), or Bay11-7082 (10 μM) for 1 h and then incubated with TNF-α (15 ng/ml) for the indicated time intervals. The levels of sICAM-1 were determined in conditioned media using an sICAM-1 ELISA kit. Data are expressed as mean±SEM of three independent experiments. ^#^*P* < 0.01, as compare to the cells exposed to vehicle **(A,D)** or TNF-α alone **(B-D). (F)** Schematic representation of signaling pathways involved in TNF-α-induced MMP-9 expression and sICAM-1 release in MC3T3-E1 cells. TNF-α stimulates two independent pathways through TNFR1/TRAF2 activates both c-Src-dependent MAPKs and c-Src-independent IKK/NF-κB pathways.

Several studies have shown that up-regulation of MMP-9 mRNA is mediated through an NF-κB-dependent pathway [35]. MMP-9 promoter also contains NF-κB binding sites [41]. To determine whether NF-κB element is essential for TNF-α-induced MMP-9 gene regulation, the MMP-9 promoter was constructed into a pGL3-Basic vector containing a luciferase reporter system (as illustrated in Figure 8A, upper part; pGL-MMP-9-Luc), which contains several putative recognition elements for a variety of transcriptional factors such as NF-κB. Next, to determine the effect of TNF-α on the MMP-9 promoter activity, cells were transfected with a pGL-MMP-9-Luc construct and then incubated with TNF-α for the indicated time intervals. As shown in Figure 8A (lower panel), TNF-α increased the MMP-9 promoter activity in a time-dependent manner. A maximal response was obtained within 10 h. The increasing of MMP-9 promoter activity stimulated by TNF-α was significantly inhibited by pretreatment with the TNFR antibody or the inhibitor of c-Src (PP1), MEK1/2 (U0126), p38 MAPK (SB202190), JNK1/2 (SP600125), or NF-κB (Bay11-7082) (Figure 8B). To further ensure that NF-κB indeed mediated TNF-α-induced MMP-9 promoter activity through binding to NF-κB element on the MMP-9 promoter region, a wild-type (WT) MMP-9 promoter mutated by a single-point mutation of the NF-κB binding site (mt-κB) was constructed (as indicated in Figure 8C, upper part), TNF-α-stimulated MMP-9 promoter activity was significantly blocked in MC3T3-E1 cells transfected with a mt-κB-MMP-9 reporter construct (Figure 8C, lower part), indicating that NF-κB binding element was required for TNF-α-induced MMP-9 promoter activity. These results demonstrated that TNF-α-induced MMP-9 promoter activity is mediated through an NF-κB binding domain of the MMP-9 promoter region in MC3T3-E1 cells.

### TNF-α-induced MMP-9 expression contributes to enhancing soluble ICAM-1 production

Previous report has shown that TNF-α induces membrane and soluble forms of ICAM-1 release by MMP-9 activity in human osteoblast-like cells [29]. Thus, we determined whether up-regulation of MMP-9 by TNF-α may contribute to a MMP-dependent release of sICAM-1, a broad spectrum MMP inhibitor GM6001, and an MMP-2/9 selective inhibitor MMP-2/9i, were used. As shown in Figure 8D (upper panel), TNF-α-enhanced sICAM-1 release in the conditioned media was attenuated by the pretreatment with GM6001 (10 μM) or MMP-2/9i (10 μM), suggesting that MMP-9 participates in TNF-α-induced sICAM-1 release. Similarly, sICAM-1 release was also detected by using a high sensitive sICAM-1 ELISA kit. The data showed that TNF-α significantly enhanced sICAM-1 release within 36 h which was significantly inhibited by the pretreatment with MMP-2/9i (10 μM), PP1 (10 μM), U0126 (10 μM), SB202190 (10 μM), SP600125 (10 μM), or Bay11-7082 (10 μM), in MC3T3-E1 cells (Figure 8D, lower part). Furthermore, we found that there was no effect on the ICAM-1 protein expression induced by TNF-α in the presence and absence of GM6001 or MMP-2/9i for 24 h (Figure 8E). Taken together, these data confirmed that up-regulation of MMP-9 is associated with the release of sICAM-1 on MC3T3-E1 cells challenged with TNF-α.

## Discussion

MMP-9 is highly expressed in osteoclasts and plays an important role in degradation of ECM. Given the role of osteoblasts in building the bone, it is somewhat counter-intuitive that they also express many matrix degrading MMPs including MMP-2, MMP-3, MMP-8, MMP-9, MMP-13, and MMP-14 [42]. It is generally accepted that locally increased levels of MMPs have been found in several osteoarticular diseases [6]. Of considerable importance in osteoarticular diseases, MMP-2 and MMP-9 can degrade and denature type I and V collagen. Most studies support the notion that TNF-α induces the production of MMP-9 in different cell types [20,38]. Several lines of evidence suggest that TNF-α treatment of cultured bone explants or cell cultures of mineralizing osteoblasts increased bone resorption and inhibited bone formation [43]. In response to inflammatory processes of bone microenvironment, MMP-9 synthesis and secretion were significantly induced by TNF-α in mesenchymal stem cells-derived osteoprogenitor, precursor of osteoblasts [13]. In this study, we established specific mechanisms by which TNF-α promotes MMP-9 expression in osteoblasts-like MC3T3-E1 cells. Based on these findings, Figure 8F depicts a model for the TNFR1-mediated activation of c-Src-dependent MAPKs (*i.e.* ERK1/2, p38 MAPK, and JNK1/2) and c-Src-independent IKK-NF-κB signaling pathways involved in TNF-α-induced MMP-9 expression and s-ICAM-1 release from MC3T3-E1 cells.

Several reports have indicated that most known responses to TNF-α are triggered by binding to one of two distinct receptors, TNFRl and TNFR2, which are differentially regulated on various cell types in normal and diseased tissues. In osteoblasts, TNF-α stimulates osteoblast differentiation through its TNFR1 receptor [44]. Recent studies have further demonstrated that TNFR1 signal transduction is mediated through the assembly of kinases, adaptors, and scaffolding proteins which also interacts with TRAF2 and IKK leading to activation of NF-κB [45]. In addition, several reports suggest that Src tyrosine kinases promote inflammatory processes under various pathologic conditions. For example, T cell protein tyrosine phosphatase interacted with TRAF2 and inactivated c-Src tyrosine kinases to selectively suppress TNF-α-induced MAPK signaling and modulate inflammatory responses [46]. However, little was known about the mechanisms of TNF-α-induced MMP-9 expression mediated through TNFR1-TRAF2-c-Src-dependent pathway in osteoblasts. Here, we hypothesized that TRAF2 and c-Src are signal transducers of TNFR1 in osteoblasts. This note was confirmed by the results indicating that TNF-α-induced MMP-9 expression was significantly blocked by TNFR antibody and c-Src inhibitor. Moreover, we used immunoprecipitation to determine the interaction among TNFR1, TRAF2, and c-Src to confirm that TNF-α induced TNFR1, TRAF2 and c-Src association. TNF-α has further been shown to stimulate the phosphorylation of c-Src which was also attenuated by c-Src inhibitor PP1 and siRNA for TRAF2. Our data were first identified that TNF-α up-regulates the interaction between TNFR1, TRAF2, and c-Src components, leading to MMP-9 expression in osteoblasts. These results suggested that TNF-α induces MMP-9 expression via TNFR1/TRAF2-mediated activation of c-Src in MC3T3-E1 cells.

Several groups of investigators have reported that TNF-α released during acute and chronic diseases activates multiple intracellular signaling cascades including the MAPKs and NF-κB signaling pathways in various cell types. Previous reports have shown that aggregation of TNFR1/TRAF2 protein complex transducer activates downstream IKKα/β-NF-κB cascade and JNK1/2 and p38 MAPK in skeletal pathologies [43]. TNF-α, a potent pro-inflammatory cytokine, has been reported to activate downstream protein kinases cascade such as MAPKs in various cells types [47]. For example, phosphorylation of p42/p44 MAPK and JNK1/2, and transactivation of NF-κB are essential for TNF-α-induced MMP-9 gene expression in A549 cells [40]. However, the activated TNFR1/TRAF2 stimulates MAPKs or NF-κB signaling pathway leading to TNF-α-induced MMP-9 expression in osteoblasts remains unclear. In this study, we found that TNF-α-stimulated ERK1/2, p38 MAPK, and JNK1/2 phosphorylation in MC3T3-E1 cells, which were attenuated by the pretreatment with PP1 or transfection with c-Src siRNA, suggesting that TNF-α stimulates phosphorylation of these MAPKs via a c-Src-dependent manner (Figure 7A and B). These data suggested that TNF-α induces MMP-9 expression is mediated through c-Src-dependent MAPKs pathway in MC3T3-E1 cells.

In addition, NF-κB is an inducible transcription factor that plays a key role in the expression of inflammatory response genes. NF-κB plays a pivotal role in bone remodeling cycle [48]. TNF-α binds its receptor to activate several intracellular signaling pathways. Aggregation of a protein complex including TRAF2 transduces the signal along the IKK/I-κB pathway leading to phosphorylation of IκB-α with liberation of the transcription factor NF-κB for nuclear entry and regulation of gene transcription [43]. In this study, our data showed that pretreatment with PP1 or transfection with siRNA of c-Src, had no significant inhibition on TNF-α-stimulated IKKα/β and p65 phosphorylation, suggesting that TNF-α-stimulated p65 phosphorylation is independent of c-Src (Figure 7A and B). Furthermore, pretreatment with the inhibitor of MEK1/2, p38 MAPK, or JNK1/2 had no effect on TNF-α-stimulated p65 phosphorylation (Figure 7C), nuclear translocation (Figure 7D), and transcriptional activity (Figure 7E), suggesting that TNF-α-stimulated p65 NF-κB activation is independent of c-Src/MAPKs in MC3T3-E1 cells. Moreover, our data showed that TNF-α stimulated IKKα/β phosphorylation (Figure 6C), suggesting that activation of IKKα/β may contribute to NF-κB activation in MC3T3-E1 cells. For the regulation of MMP-9 promoter, we also demonstrated that TNF-α-stimulated activation of MMP-9-promoter-luciferase activity was inhibited by pretreatment with TNFR1 antibody, PP1, U0126, SB202190, SP600125, or Bay11-7082 (Figure 8B). We further confirmed that NF-κB binding site within MMP-9 promoter is important for TNF-α-induced MMP-9 expression by transfection with a MMP-9 promoter constructed with NF-κB binding site mutation (Figure 8C), indicating that NF-κB binding domain is required for MMP-9 promoter activation by TNF-α in MC3T3-E1 cells. These data suggested that TNF-α-stimulated MMP-9 gene expression is mediated through NF-κB-mediated up-regulating MMP-9 promoter activity, and which involved TNFR1, c-Src-dependent MAPKs (*i.e.* ERK1/2, p38 MAPK, and JNK1/2) and c-Src-independent IKK/NF-κB pathways. MAPKs are serine/threonine protein kinases, which contribute to several cellular pathophysiological responses through regulation of their downstream molecules including transcription factors. Previous studies have indicated that TNF-α induces MMP-9 expression via a MAPK-dependent activation of NF-κB or AP-1 in several cell types [37,39,49]. Here we demonstrated that TNF-α-induced MMP-9 expression is mediated through a MAPK-independent NF-κB pathway. Next, we also suggested that TNF-α might induce MMP-9 expression via a MAPK-dependent AP-1 pathway in MC3T3-E1 cells. These results will be confirmed in the future.

In bone metabolism, ICAM-1 importantly mediates cell-cell adhesion of osteoblasts and osteoclast precursors, thereby facilitating osteoclast differentiation and bone resorption [25,50]. Osteoblasts regulate osteoclast recruitment of bone resorption through RANKL and ICAM-1. In bone diseases, blockage of the interaction between TNF-α and sICAM-1 may inhibit not only inflammation in the joints but also bone resorption by suppressing the osteoblast-mediated formation of osteoclasts [24]. Treatment of osteoblasts with the chemical inhibitor of MMP-9 activity, a proteolytic enzyme involved in ICAM-1 cleavage, displayed a significant decrease of TNF-α-induced sICAM-1 release [29]. Finally, we examined a functional consequence of TNF-α-induced MMP-9 expression in mature osteoblasts by sICAM-1 determination. In this study, we demonstrated that TNF-α induces MMP-9 up-regulation that promotes sICAM-1 release into the conditioned media (Figure 8D), but no effect on the ICAM-1 protein level (Figure 8E). Our results are consistent with previous report indicating that TNF-α-increased MMP-9 activity may act on mICAM-1 resulting in sICAM-1 release. These findings concerning TNF-α-induced MMP-9 expression and sICAM-1 release in MC3T3-E1 cells imply that TNF-α might play a critical role in the development of bone inflammatory process.

## Conclusions

Our study provides new insights into the mechanisms by which TNFR1/TRAF2 activates both IKKα/β-NF-κB and c-Src-ERK1/2, p38 MAPK, and JNK1/2 pathways may be associated with MMP-9 expression in osteoblasts-like MC3T3-E1 cells (Figure 8F). In addition, our findings indicated that increased MMP-9 may contribute to mICAM-1 protein cleavage on the surface of ostoblasts leading to sICAM-1 release. Targeting MMP-9 inhibition by pharmacological approaches could have clinical interventions in the treatment of bone loss diseases, such as arthritis and aseptic loosening. Moreover, the capacity of MMP-9 to selectively prevent production of sICAM-1 may be useful for the development of novel therapeutic approaches relevant for the management of bone inflammation.

## Abbreviations

TNF-α: Tumor necrosis factor-α; MMP-9: Matrix metalloproteinase-9; MC3T3 cells: Mouse osteoblast-like cells; α-MEM: Minimal essential medium-alpha; FBS: Fetal bovine serum; ECL: Enhanced chemiluminescence; BCA: Bicinchoninic acid; PBS: Phosphate-buffered saline; BCA: Bicinchoninic acid; TNFR1: TNF receptor l; TRAF2: TNF receptor-associated factor 2; MAPKs: Mitogen-activated protein kinases; ERK: Extracellular-regulated protein kinase; JNK: c-Jun-N-terminal kinase; sICAM-1: Soluble intercellular adhesion molecule-1; IP: Immunoprecipitation; siRNA: Small interfering RNA; RT-PCR: Reverse transcription-polymerase chain reaction; NF-κB: Nuclear factor-kappa B.

## Competing interests

The authors declare that they have no competing interests related to this work.

## Authors’ contributions

Conceived and designed the experiments: CLT, HLH, WCC, and CMY. Acquisition of data: CLT, PLC, and WCC. Analysis and interpretation of data: CMY. Drafting the article: CLT, HLH, WCC and LDH. Revising the article for important intellectual content: HLH and CMY. Final approval of the version to be published: CLT, HLH, PLC, WCC, LDH, and CMY.
