# Erratum to: TNF-α induces MMP-9 expression and soluble ICAM-1 release via TRAF2, c-Src, MAPKs and NF-κB in osteoblast-like MC3T3-E1 cells

**DOI:** 10.1186/s12929-015-0119-1

**Published:** 2015-03-20

**Authors:** Tsai Chia-Lan, Wei-Chung Chen, Hsi-Lung Hsieh, Pei-Ling Chi, Li-Der Hsiao, Chuen-Mao Yang

**Affiliations:** Department of Physiology and Pharmacology and Health Ageing Research Center, College of Medicine, Chang Gung University, Tao-Yuan, Taiwan; Department of Nursing, Division of Basic Medical Sciences, Chang Gung University of Science and Technology, Tao-Yuan, Taiwan; Department of Physiology and Pharmacology, Chang Gung University, 259 Wen-Hwa 1st Road, Kwei-San, Tao-Yuan Taiwan

## Erratum

It has come to our attention that Figures 2F and 7A in our article [1] are the same as Figures 1E and 6A in our previously published article in Bone [2] and there is some text overlap in the Methods section. Both papers approached the various intracellular signalling pathways involved in TNF-α-induced MMP-9 expression in osteoblast-like MC3T3-E1 cells, which shared common signalling molecules such as Src and p42/p44 MAPK. The images presented come from the same experiment described in both articles. Due to the similar approach used in each case there is text overlap in the Methods section. We apologize for this overlap and any inconvenience caused.
